# Factors Influencing Temperature Measurements from Miniaturized Thermal Infrared (TIR) Cameras: A Laboratory-Based Approach

**DOI:** 10.3390/s21248466

**Published:** 2021-12-18

**Authors:** Quanxing Wan, Benjamin Brede, Magdalena Smigaj, Lammert Kooistra

**Affiliations:** 1 Laboratory of Geo-Information Science and Remote Sensing, Wageningen University & Research, Droevendaalsesteeg 3, 6708 PB Wageningen, The Netherlands; benjamin.brede@wur.nl (B.B.); magdalena.smigaj@wur.nl (M.S.); lammert.kooistra@wur.nl (L.K.); 2 Helmholtz Center Potsdam GFZ German Research Centre for Geosciences, Section 1.4 Remote Sensing and Geoinformatics, Telegrafenberg, 14473 Potsdam, Germany; 3 School of Geography, Politics and Sociology, Newcastle University, Newcastle upon Tyne NE1 7RU, UK

**Keywords:** UAV, thermal infrared, FLIR, radiometric, calibration, temperature, non-uniformity correction, stabilization, sensor temperature, ambient environment

## Abstract

The workflow for estimating the temperature in agricultural fields from multiple sensors needs to be optimized upon testing each type of sensor’s actual user performance. In this sense, readily available miniaturized UAV-based thermal infrared (TIR) cameras can be combined with proximal sensors in measuring the surface temperature. Before the two types of cameras can be operationally used in the field, laboratory experiments are needed to fully understand their capabilities and all the influencing factors. We present the measurement results of laboratory experiments of UAV-borne WIRIS 2nd GEN and handheld FLIR E8-XT cameras. For these uncooled sensors, it took 30 to 60 min for the measured signal to stabilize and the sensor temperature drifted continuously. The drifting sensor temperature was strongly correlated to the measured signal. Specifically for WIRIS, the automated non-uniformity correction (NUC) contributed to extra uncertainty in measurements. Another problem was the temperature measurement dependency on various ambient environmental parameters. An increase in the measuring distance resulted in the underestimation of surface temperature, though the degree of change may also come from reflected radiation from neighboring objects, water vapor absorption, and the object size in the field of view (FOV). Wind and radiation tests suggested that these factors can contribute to the uncertainty of several Celsius degrees in measured results. Based on these indoor experiment results, we provide a list of suggestions on the potential practices for deriving accurate temperature data from radiometric miniaturized TIR cameras in actual field practices for (agro-)environmental research.

## 1. Introduction

### 1.1. Context and Background

From the 1970s on, satellite-based thermal infrared (TIR) remote sensing has been applied widely to regional-scale investigations, for instance, hydrological modeling [1,2,3], mineral exploration [4,5], urban thermal environment [6], and forest fire detection [7]. In this century, the technological development in easy-to-use low-cost miniaturized TIR cameras has enabled their application on Unmanned Aerial Vehicles (UAV) in addition to proximal platforms for local-scale studies [8,9,10,11]. UAVs have emerged as powerful platforms for facilitating the acquisition of data on high temporal and spatial scales, extending current remote-sensing-based monitoring capabilities owing to their flexibility in the exact timing of flight missions and adaptive flight altitude [12]. The dedicated uncooled TIR sensors are light-weight, low in power consumption, and have a high degree of compatibility with various widely used UAV platforms [10]. The other option—cooled sensors—are for many UAV platforms too heavy to be mounted, although they can provide higher accuracy measurements [13,14]. The combination of UAV and proximal thermal imaging, whereby proximal measurements are used for calibration purposes, is a promising solution for operationalizing UAV thermal surveys. This approach has started to be adopted in a variety of agricultural applications, e.g., for crop water stress (CWS, or water-deficit stress) detection [15,16,17,18]. The detection of CWS’s development requires detecting subtle temperature changes, which might not be as crucial for other applications. The capability of capturing subtle changes needs a good understanding of the structure and work process of TIR cameras and error sources. Moreover, identifying the spatial and temporal distribution of CWS is important to plan irrigation schemes, and therefore saving water use and ensuring food production. To achieve that, a series of CWS indices based on canopy temperature have been developed and used for indicating the crop water status. These indicators serve for in-field CWS monitoring together with ground measurements of crop physiological indices (e.g., soil water content, stomatal conductance (*g_s_*), and transpiration rate (*t_r_*)) [19,20,21,22].

The performance of uncooled TIR cameras can be affected by multiple sources of errors. Firstly, the choice of uncooled TIR detectors determines the cameras’ performance. These detectors can be divided into pyroelectric detectors, thermopile detectors, microbolometer detectors (hereinafter referred to as *bolometers*) [23]. The bolometer’s technical route has become the mainstream technical direction [24]. The most used sensitive materials for the bolometer are vanadium oxide (VO_x_) and amorphous silicon (α-Si) [25,26,27]. For both, the detection sensitivity to infrared signals is lower than that of cooled detectors. Their ability to convert infrared radiation into temperature changes is weaker and therefore subtle temperature differences are difficult to distinguish. Secondly, the miniaturization process restricts the accuracy and sensitivity of these TIR sensors [28]. In practice, the miniaturized TIR camera’s temperature reading and its components—the gain (sensitivity) and offset of each microbolometer often change with the sensor (focal plane array, FPA) temperature, since its core does not include a cooling system [23]. The above-mentioned temperature drifts can be compensated by performing non-uniformity corrections (NUCs) with high frequency [28,29,30]. This temperature deviation increases during UAV flight campaigns because of the rapid changes in the ambient environmental conditions [31]. Thirdly, the bolometer also receives the thermal radiation emitted from the inside of the camera, which may be larger than the thermal radiation received by the target object, resulting in a poor signal-to-noise ratio in the acquired TIR imagery [32]. Lastly, a series of external influencing factors that can also influence the performance of TIR cameras is the emissivity of the target object, the measuring distance between the sensor and the target object, the atmospheric conditions, and so forth [33]. 

### 1.2. Practices for Deriving Temperature Data in Field Situations

In the domain of optical engineering, in-depth research has been conducted continuously on the working principle and radiometric calibration methods of miniaturized TIR sensors. The theoretical research has been widely practiced in various applications, which gives insights into the internal radiative transmission process of the sensor (as one limiting factor), other limiting factors of accurate measurements, and corresponding correction algorithms [8,16,34,35,36]. In real-world applications, whether the miniaturized TIR sensor can maintain the accuracy level has become a major challenge. Accordingly, the radiometric calibration of the sensor is a necessity, and then the quantitative correlation between the temperature of the measured object and the raw value of each FPA unit can be determined [37]. 

The calibration process can be challenging to implement for users. As one manufacturer stated, the follow-up factors affect the temperature measurement accuracy of miniaturized TIR sensors, including surface characteristics, atmospheric interference, and imaging system [38]. First of all, it is necessary to apply emissivity correction to the blackbody measurements [39]. Emissivity indicates the surface characteristics of a certain material. Real-world blackbodies have lower emissivity, although a blackbody is a concept of an ideal emitter of which the emissivity should be one. Then, the inclusion of all the influencing factors for eliminating temperature dependency effects is still problematic [37]. The above-mentioned “atmospheric interference” refers to the influence of environmental factors on the temperature measurement process, and the “imaging system” can be understood as the influence of the sensor’s intrinsic characteristics. For miniaturized radiometric TIR sensors, the calibration algorithm of its supporting data analysis and processing software usually takes into account the main influencing factors of the sensor and the ambient environment. As the specific radiometric calibration specifications implemented by the manufacturer are proprietary, it is hard for users to determine the contribution of the aforementioned influencing factors to errors in the measurement [40,41,42]. Real-time FPA temperature needs to be regarded as one of the core parameters to obtain in the radiometric calibration process [39]. When the thermal radiation generated by different parts of the camera (internal, lens, and other optical components) is fully considered, the corresponding gain and offset values of each microbolometer can be determined and microbolometers’ non-uniform responses can be corrected [25,37]. Unfortunately, it is difficult to obtain FPA temperature for many manufacturers’ equipment.

Previous research tested the raw output of miniaturized TIR sensors under constrained conditions. This study provides insight into the accurate temperature derivation. Many laboratory calibration methods of the sensors have been made available despite the unavailability of sensor temperature [37,43,44]. In summary, relevant research was first to obtain a large number of measurement samples by controlling variables and repeating sessions in an indoor environment and then calibrating the measurements, aiming to achieve high precision and consistency. Successfully developed calibration models in the laboratory environment were eventually applied to the field tests [41,45,46,47]. The achieved consensus is that: the laboratory calibration can usually obtain accurate calibration results (±0.5 °C accuracy). However, during field tests, the actual performance of the chosen calibration methods is significantly worse. The uncertainty can increase easily up to several degrees (>5 °C) since the original relationship between the FPA temperature and the measurements are no longer valid [10,39,48]. These studies have developed or adapted methods that minimize multiple factors’ influences on camera outputs with no elaborated discussion on the separate impacts [37]. Kelly et al. [37] reminded upcoming researchers of the extent to which individual influencing factors can challenge deriving accurate measurements for uncalibrated TIR cameras. This work aims to separate the influence of multiple factors on the camera readings of the calibrated TIR cameras and respectively quantify the contribution of each to the degree of deviation of the measured value, to guide the corresponding adjustments in the field practices. Two representative radiometric TIR camera models (UAV-borne and handheld) were used to test the influence of various factors on camera performances. Several studies [8,37,43] had tested a single TIR camera’s performance in the laboratory environment, however, the results may be affected by the model selection. What needs to be explored is whether different types of TIR cameras have similar performance under the same parameter settings. Whether the phenomena existing in non-radiometric cameras can be observed in radiometric cameras is also yet to be established. 

### 1.3. Research Objectives

The primary goal of the research is to explore the feasibility of applying different types of miniaturized TIR cameras to field practices requiring high accuracy, such as crop water stress mapping. The controlled-environment experiment results will be used to put forward practical recommendations towards the design of field tests, for obtaining high-precision in field measurements.

Specifically, the influence of the intrinsic characteristics of the TIR camera on the accurate temperature measurement has been tested based on the following research questions (RQs): a. How long does it take for the miniaturized TIR cameras to stabilize after being switched on? b. How do the periodic NUCs affect the temperature measurements? c. Will changes in sensor temperature have a significant impact on the measured temperature values of the UAV-mounted and handheld TIR cameras? Besides, the influence of environmental factors has also been tested: d. Will the measuring distance have a strong effect on the measured temperature values of UAV-mounted and handheld TIR cameras? e. How do changes in wind and radiation affect the temperature measured by a UAV-mounted TIR camera?

## 2. Materials and Methods

### 2.1. Materials

For this study, we used a WIRIS 2nd GEN radiometric TIR camera designed specifically for the use on a UAV, and a FLIR E8-XT handheld camera only for reference measurements on the ground. Both of these miniaturized TIR cameras used a core equipped with a vanadium oxide (VO_x_) microbolometer FPA, and their working principle was comparable to that of other camera models [27]. Therefore, the practices using these cameras are of significant reference to the tests with other models. 

Workswell WIRIS 2nd GEN, a UAV-mounted TIR camera (Workswell design and manufacturing company, Praha, Czech Republic)—henceforth referred to as WIRIS—can capture calibrated images, which means that the measured object’s radiant temperature can be recorded in high resolution directly from the output data set. The second camera—FLIR E8-XT handheld TIR camera (FLIR Systems, Inc., Wilsonville, OR, USA) does the same but has a lower resolution. For FLIR E8-XT, the FLIR ResearchIR thermal analysis software was adopted for camera system command and control, enabling high-speed data recording. The specifications of adopted cameras’ features are listed in Table 1.

### 2.2. Experimental Set-Up of Laboratory Experiments

#### 2.2.1. General Description

The main research method is to design a series of experiments by controlling variables in a laboratory environment to determine the influence of the ambient environment and the TIR camera’s intrinsic characteristics on the accuracy of temperature measurement. Upon all the key parameters and environmental factors being adjusted and quantified, the experimental design of the field tests can be optimized by evaluating the laboratory results.

Four experiments have been conducted for testing the response characteristics of TIR sensors to thermal radiation signals. Two of the experiments were used to explore the influence of the intrinsic characteristics of TIR cameras on the temperature measurements: (a) assessing the stabilization time of TIR cameras, (b) generating calibration curves by measuring the cameras’ responses to different sensor temperatures, indirectly achieved by adjusting the ambient temperature. The remaining sessions aimed to explain the influence of ambient environmental factors on accurate measurements: (c) the effect of the change in the thickness of the atmospheric layer between the sensor and the target on the measured temperature, caused by the distance variation between the camera and the blackbody, (d) assessing wind and heating effects on temperature outputs of cameras. All sub-experiments in this research used two blackbody calibrators (models KBB 35 and KBB 55 from Kleiber Infrared GmbH, Germany; emissivity = 0.98 ± 0.004, temperature uncertainty = 0.4 °C for T_a_ = 10–30 °C and 0.6 °C for T_a_ = 0–10 °C, reproducibility = 0.2 °C, stability = 0.1 °C, uniformity = 0.2 °C for the 45-mm-diameter central area) with fixed temperatures of 35 °C and 55 °C to compare the performance of adopted cameras against the target objects.

**Figure 1:**
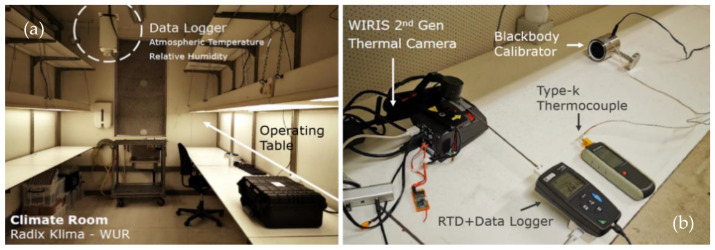
General introduction of the experimental design.

The introduction to the laboratory and instruments can refer to Figure 1. During experiments, the housing (surface) temperature of the camera was recorded at 1 Hz with a PT100 resistance temperature detector (RTD) (SP14, Chauvin Arnoux Group, Paris, France) and a data logger (CA 1823, Chauvin Arnoux Group, Paris, France), as shown in Figure 1b. In an environment with its temperature ranging from −40 °C to 450 °C, the measurement accuracy of this RTD can reach 0.15 °C. A weather monitor (ImagDlo, the Netherlands) provided automatic monitoring data of indoor meteorological elements, including air temperature (T_a_) and relative humidity (RH), every 10 min. Table 2 provides details of the experimental set-ups. Regarding the output data formats, raw data was captured as 16-bit TIFF images for WIRIS, while the storage was in sequential (SEQ) digital format for FLIR E8-XT. 

#### 2.2.2. Stabilization Time

To test the amount of time required for the TIR cameras to output stable readings, images mainly containing the blackbody calibrator signal were captured at regular intervals for a period of up to 3 h (Table 2), a time period that can typically easily be supported by UAV batteries. The long waiting time on the ground ensured that there was enough time to stabilize the camera signal before take-off, so the testing time for this type of camera in the indoor experiments was longer. By contrast, the battery life of handheld TIR cameras was shorter. Therefore, the goal was to understand whether the measured temperature could reach a steady-state under strict time constraints for handheld cameras. In WIRIS sub-experiments, two different NUC execution frequencies were applied (2 and 30 min) to explore automatic NUCs’ influence on temperature measurements. 

#### 2.2.3. Sensor Temperature

The experimental setup for testing the sensor temperature’s influence was inspired by the standardized calibration procedure [37,41]: using a standard heat source (blackbody calibrator) with a constant emissivity, starting heating the blackbody in a fixed temperature step, and then using a TIR sensor to measure the blackbody temperature. The temperature readings are compared with the blackbody temperature, and a calibration curve is formed after collecting multiple samples. Next, when the ambient temperature has been adjusted, it is possible to test whether the response characteristics of a specific sensor are varying under different ambient temperatures. Since this study used a blackbody radiator of which the temperature could not be adjusted, it was not possible to obtain a standard blackbody calibration curve at multiple blackbody temperatures. On the premise of retaining similar experimental purposes, our solution was to change the major variable from blackbody temperature to ambient temperature. Even though such an experimental setting cannot reflect the multiple linear regression between the measured temperature, ambient temperature, and blackbody temperature, it can indirectly indicate how the sensor’s temperature affects the measurements instead. The sensor temperature is affected by the environment and cannot be maintained at a constant temperature for uncooled TIR cameras. Therefore, by adjusting the ambient temperature, we studied the effect of sensor temperature on camera readings before temperature dependence corrections.

The adjustment of ambient temperature in the climate room (the embedded climate box was manufactured by Hoogendoorn, size: 5 × 3 m, located in Radix Klima, Wageningen University and Research) followed two constraints. Firstly, temperature adjustments had to be done on an hourly basis, and the step of each adjustment could not exceed 5 °C. Secondly, the range of temperature change referred to the atmospheric temperature that might be experienced during the growth period of the local major crops. Therefore, we referred to the historical temperature data of Wageningen from May to October. During this period, the monthly average of day temperature in this region could reach up to 22 °C in summer, and the lowest monthly average night temperature was 6 °C in winter. Accordingly, we extended the temperature range and set the change of ambient temperature in steps from 5 to 25 degrees in the program, with the intermediate points at 10 °C, 14 °C, 18 °C, 20 °C, and 22 °C, respectively. In effect, there was no abrupt change of ambient temperature as it took several minutes for the temperature of the whole climate room to reach the set value.

#### 2.2.4. Sensor–Object Distance

Being restrained by the size of the climate room, the measuring distance between the TIR camera and black body ranged from 0.5 m to 4 m (Table 2). For a given measuring distance, images were captured every 5 s and lasted for 20 min. Specifically for WIRIS, the execution of NUCs was enabled every 30 min, and the start time of each round of measurements was set to 5 min after the last NUC. Therefore, it was ensured that no NUCs were executed during the measurement periods for both sensors.

#### 2.2.5. Wind and Heating Effects

In reality, the ambient environmental conditions that a UAV-mounted TIR camera would experience in the air are constantly changing to varying degrees during flight campaigns. A fan and a thermo-heater gun were respectively used to simulate the influence of wind and solar radiation in the natural environment on the temperature measurement result of WIRIS. (a) For the fan experiment: the camera was kept 0.5 m away from the blackbody. A fan was placed also 0.5 m apart from the side of WIRIS in the direction perpendicular to the camera-blackbody line. After WIRIS had been turned on for 1 h, the fan was turned on at its wind speed level 1, specifically 2.7–3.1 m/s. The wind blew towards the camera continuously for 30 min and at the same time the camera recorded blackbody images every 5 s. After the first round of wind experiments, there was a control period of 60 min. The determination of this length of time was based on the results of the “stabilization time” experiment (refer to Section 2.2.2). Afterward, the second wind level (2.9–3.3 m/s) was applied, followed by another 60-min control period. At last, the experiment was repeated with the wind level 3 at speeds between 3.1 and 3.7 m/s for 30 min. (b) For the thermo-heater gun experiment: the relative positions of all instruments were the same as in the previous section. After WIRIS had been warmed up for 1 h, the thermo-heater gun was turned on at its level 1 (100 °C). The hot air blew towards the camera for 30 min and the camera imaged the blackbody every 10 s simultaneously. After the next control period of 30 min, the heating-up test continued with a higher level (200 °C thermal wind) for 15 min. The duration of this experiment was shortened to avoid damage to the camera due to overheating.

#### 2.2.6. Data Analysis

In the data processing stage, the blackbody extent was determined as the region of interest (ROI), and only the measured temperature values within this range were analyzed. Extra attention was paid to accurately locating the area corresponding to the blackbody in the TIR images during “sensor–object distance” sub-experiments. As the measurement distance increased, the proportion of the corresponding area in the images was significantly reduced. Therefore, using the region of interest (ROI) to accurately frame the blackbody area required more effort. This step was realized in combination with the reference values in the background and overlapping with the corresponding visible (VIS) images. To derive statistical analysis results from acquired TIR images, ImageJ [49] was used to enable batch processing of WIRIS data, while FLIR Research Studio was used to analyze the encrypted SEQ input data from FLIR E8-XT. As for statistical indicators, the average value was for checking the overall situation of each blackbody image, and the standard deviation (STD) served for analyzing the degree of difference in the response of different FPA units of TIR cameras to radiation. 

## 3. Results

### 3.1. Measured Temperature’s Stability over Time

**Figure 2:**
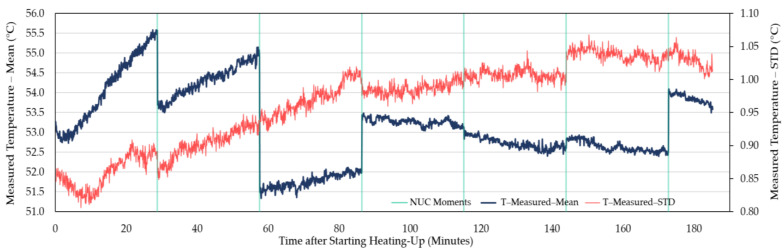
The original time series curves of WIRIS camera observations. Dark blue thick curve: Readings of the average measured temperature over time when imaging a round-shape blackbody calibrator at 55 °C, as recorded by WIRIS over 3 h after switching on the camera. During the operation of the camera, the emissivity of the measured object was adjusted from the default value of 0.95 to 0.98, being consistent with that of the adopted blackbody calibrator (Ε_BB_ = 0.98). The thin red curve represents the development of the standard deviation (STD) of the temperature readings. The non-uniformity corrections (NUCs) every 30 min are shown as green lines.

**Figure 3:**
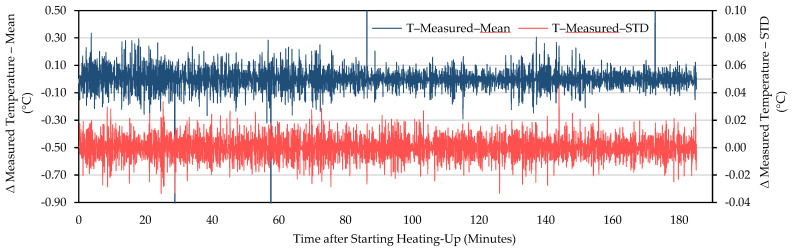
The first-order difference series curves of the WIRIS camera readings. The dark blue curve represents the first-order differential time series data of the observed mean. The red curve is the first-order difference time series of the STD of the observations.

**Figure 4:**
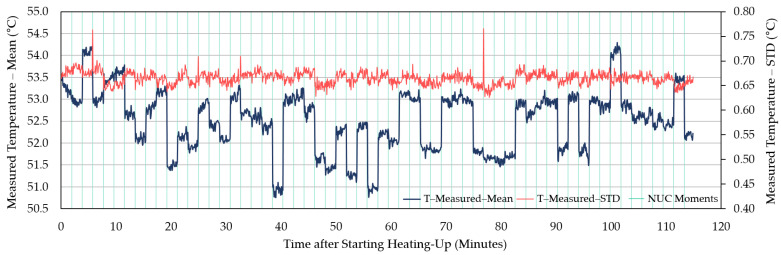
The measured average temperature of the blackbody calibrator (55 °C) area, as recorded by WIRIS 2nd GEN over 2 h after switching on the camera. The STD of the measured temperature is also included. In this session, the NUCs were enabled every 2 min.

**Figure 5:**
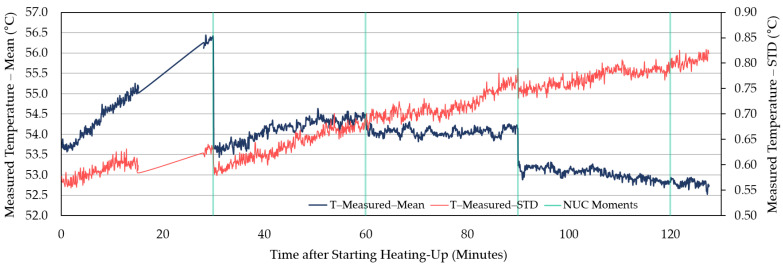
The measured average temperature of the blackbody (55 °C) area, as recorded by WIRIS 2nd GEN over 2 h after activation. The STD of the measured temperature is also included. In this session, the automatic NUCs were enabled every 30 min. This figure is used as a comparison with Figure 4 to visualize the influence of NUC frequency under the same experimental conditions. Due to a malfunction, no data was recorded between 17 and 28 min.

**Figure 6:**
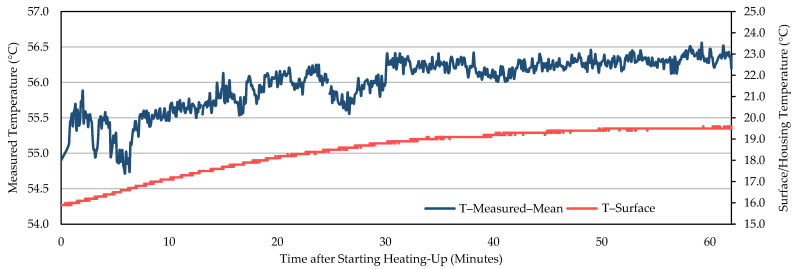
The measured average temperature of the blackbody calibrator (55 °C) area, as recorded by FLIR E8-XT over 1 h after switching on the camera. The housing temperature’s development of the camera is also depicted.

Being judged by a steady decreasing trend after the slower increase in the original curves and the starting point of the decreasing trend in the first-order difference series curves, it took about 60–90 min for the measured temperature of WIRIS to reach stability (for example, Figure 2 and Figure 3). When the measurements stabilized, an underestimation was revealed. The measured temperature was at least 1 °C lower than the actual value. Similar trends in between NUCs were observed in other repeated experiments (Figure 4, Figure 5 and Figure S1). For FLIR E8-XT, significant temperature shifts were observed over the first 30 min (Figure 6 and Figure S2). In contrary, an overestimation of approximately 0.5 °C (35 °C blackbody group) or 1.0–1.5 °C (55 °C blackbody group) was revealed when the measured temperature reached its stability.

The measurement results showed that the effect of NUC on the measured temperature was irregular for both applied frequencies. The direction and extent of signal adjustment caused by NUC were both random. From the camera activation to a long time after the completion of the warm-up period, the measured temperature has shown an increasing trend between every two NUC executions (Figure 2, Figure 4, and Figure 5). The end of the increasing trend is at approximately between 60 and 90 min for different experimental groups. After the “tipping point”, the observation data showed a downward trend in between NUCs. With frequent NUCs, the sensor corrects the gain and offset of each pixel more often. It means that the thermal stabilization reached after each NUC is difficult to break. In this case, the difference in response between different pixels is difficult to expand over time. In addition, the differences in the degree of response of different pixels (referred to STD) can be maintained at the same level. Based on the results of this session, this study applied a “stabilization phase” of a specific length of time to stabilize the temperature measurements in the following experiments. For WIRIS and FLIR E8-XT, the length of this phase was 60 min and 30 min, respectively. It means that the subsequent experimental data collection all started after the camera had been turned on for the above-mentioned time. 

### 3.2. Response to Ambient Temperature Adjustments

**Figure 7:**
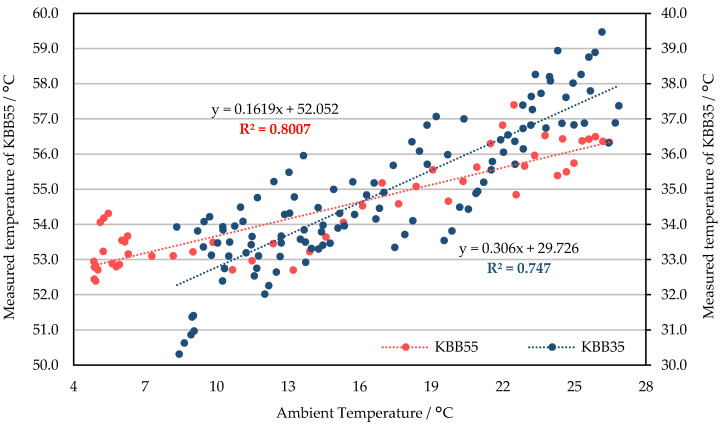
The correlation between the temperature measured by WIRIS 2nd GEN and the ambient temperature for blackbodies at 35 °C (KBB35) and 55 °C (KBB55). P-value was smaller than 0.001 for both.

**Figure 8:**
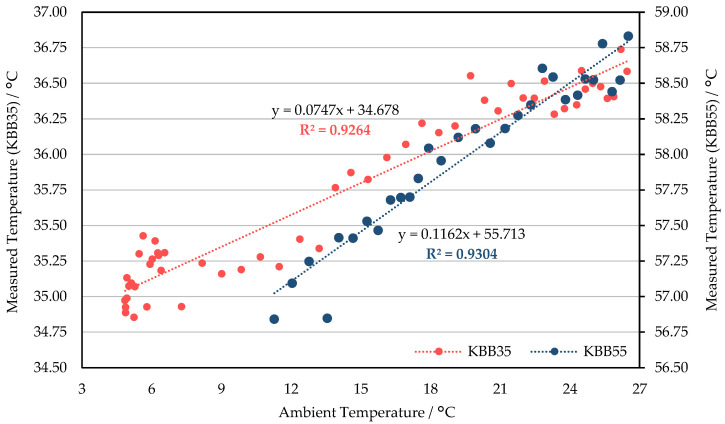
The correlation between the measured temperature by FLIR E8-XT and the ambient temperature. This session was conducted at the blackbody temperature of 35 °C (KBB35) and 55 °C (KBB 55), separately. P-value was smaller than 0.001 for both.

There was a clear linear positive correlation between the measured temperature and the ambient temperature for both TIR cameras (Figure 7 and Figure 8). Specifically for WIRIS: when targeting the 35 °C blackbody, the measured temperature increased by more than 0.16 °C for every degree the ambient temperature increased. When imaging the 55 °C blackbody, the rate of increase in the measured temperature was nearly doubled (89% higher compared to the 35 °C group). The frequent NUCs resulted in drifts in the measured temperature for any given ambient temperature. Thus, the correlation between ambient and measured temperature was negatively influenced (Figure 7). For FLIR E8-XT, the measured temperature’s correlation to ambient temperature was stronger than that of WIRIS (Figure 8). The measured temperature’s increment with the ambient temperature was greater for the 55 °C blackbody experiment.

As shown in Figure 7, the temperature measurements using WIRIS are the closest to the blackbody temperature (55 °C) when the ambient temperature is in the range of 16 °C to 18 °C. The measured temperature was underestimated (overestimated) when the ambient temperature was below (above) this interval. According to the linear regression equation of the 35 °C group, assuming that the change in ambient temperature reaches 20 °C, the corresponding measured temperature variation can exceed 5 °C (Figure 7). Compared to WIRIS, the temperature data measured by FLIR E8-XT was closer to the true value in the lower ambient temperature ranges. When the set value of the ambient temperature was within the atmospheric temperature range during the growing seasons of common crops in Wageningen (approximately 6–22 °C), the measured temperature was very likely to be overestimated (Figure 8). 

### 3.3. The Effect of Measuring Distance on the Measured Temperature

**Figure 9:**
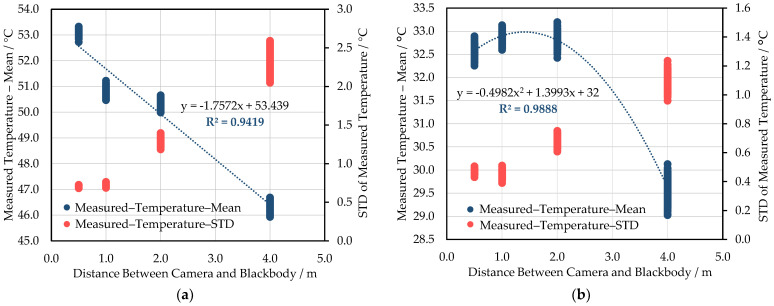
The correlation between the measured temperature by WIRIS and the measuring distance. These sessions were conducted at the blackbody temperature of 55 °C (**a**) and 35 °C (**b**). The STD of temperature observation is also included in the charts above.

**Figure 10:**
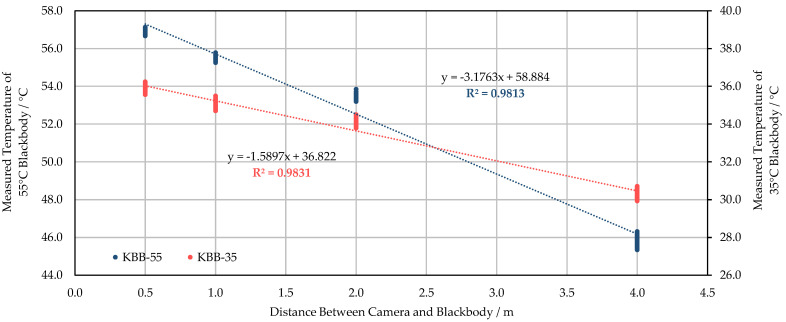
The correlation between the temperature observations measured by FLIR E8-XT and the measuring distance at the blackbody temperature of 35 °C and 55 °C.

Except for the 35 °C blackbody session of WIRIS (Figure 9b), all other tests have shown a strong negative linear correlation between the measuring distance and the measured temperature (Figure 9a and Figure 10). As can be seen from Figure 9a, as the measuring distance is extended from 0.5 m to 4 m, the deviation of temperature reading from the initial value reaches approximately 6 °C. Meanwhile, the degree of dispersion of the measured data in the blackbody extent also increases significantly as the measuring distance increases. This was affected by the sharp decrease in the number of pixels of the target object. According to Figure 9b, when the camera is moved from 0.5 m to 2 m away from the blackbody, the measured temperature does not show a downward trend. At a distance of 4 m, the measured temperature drops sharply by more than 3 °C. For FLIR E8-XT tests, it is obvious that the measured temperature decreases with the increase of distance in Figure 10. The change in observations caused by the position variance can reach about 5–10 °C.

### 3.4. Wind and Heating Up Effects on Camera’s Response

**Figure 11:**
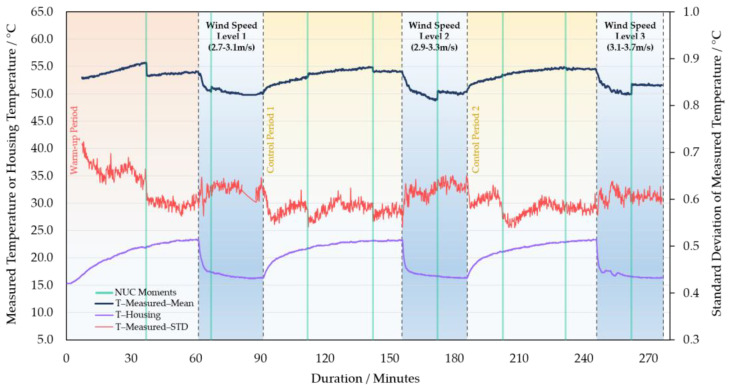
Simulation of the effect of wind on WIRIS while imaging a blackbody at 55 °C with three levels of wind speeds (lowest—2.7–3.1 m/s, medium—2.9–3.3 m/s, highest—3.1–3.7 m/s). One-hour control periods were set between wind tests. The housing temperature’s development (of the camera) and the STD of the measured temperature are also included.

**Figure 12:**
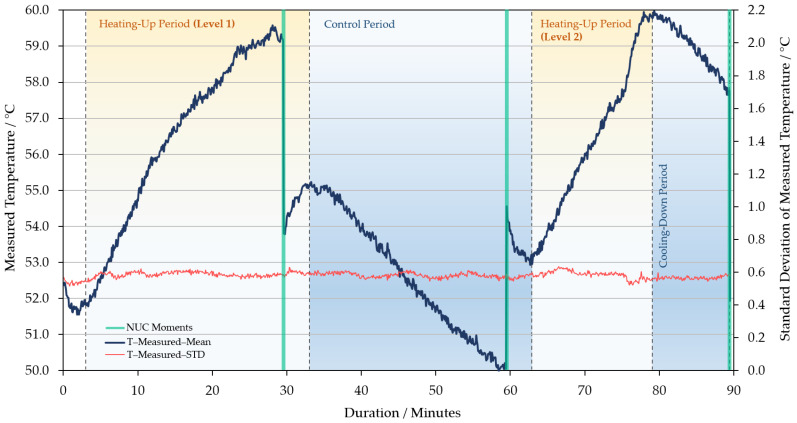
Simulation of the effect of (radiative) heating on WIRIS by a thermo-heater gun, while imaging a blackbody at 55 °C. A control period was set between wind tests. The STD of the measured temperature values is also included. The housing temperature is not available for this session.

This part of the experiment aims to simulate the effects of wind and solar radiation experienced by WIRIS when it is mounted on a UAV in the fields. During the control period of wind tests, the residual effects of the previous wind speed level on the measured temperature gradually weakened (Figure 11 and Figure S3). When the fan restarted, the measured temperature and camera housing (surface) temperature of the instrument responded quickly to the wind again. The response level of both sets of temperature measurements levelled off after 45 min (Figure 11 and Figure S3). The experiment with the 55 °C blackbody showed that different wind speeds had similar effects on the temperature measurements and housing temperature (Figure 11). In the other group using the 35 °C blackbody, a higher wind speed level brought greater impact on the temperature observations–see Supplementary Materials (Figure S3). The response difference of FPA units remained stable in the wind test. Similar to wind tests, the heating-up treatments also made a big difference to the measured temperature (Figure 12). In the process, the increase in the measured temperature could achieve higher than 7 °C. However, it was not possible to determine how long it would take for the thermal effects on temperature observations to be maximized, because the duration of the heating treatments was not long enough. 

## 4. Discussion

This research explored the feasibility of applying different types of miniaturized TIR cameras to field practices requiring high accuracy. Cameras’ performances were observed by implementing the controlled-environment experiments in a climate room. 

In terms of the time required to reach a stable state, there were differences in the performance of different TIR camera models. In the repeated experiments with two cameras in this study, the stabilization time ranged from approximately 60 to 90 min (Figure 2, Figure 4, and Figure 5, and Figure S1 in the supplementary materials). The increasing trend gradually weakened until the end of this warm-up period and the temperature drifted downward at the pace of 0.5 °C every 20–30 min afterward (Figure 2). In addition, the corresponding criteria for judging whether the measured value reached a steady-state were also different. For WIRIS, the automated NUC’s influence complicated the judgment. Images at different moments contained different degrees of NUC influence. The reason was that after each NUC the measured temperature drifted continuously until the next execution. Simultaneously, the last NUC’s correcting effect diminished. For FLIR E8-XT, the measured value could fluctuate drastically up to more than 2 °C within 10 min after the camera was turned on (Figure 6). The measured value rose gradually after a steep drop and then stabilized. No significant trend developed in the signal. Such variations in signal readings caused by the warm-up process of sensors have also been found in studies using different camera models. Kelly et al. [37] observed a similarly dramatic shift using a non-radiometric TIR camera—FLIR Vue Pro 640, in which the signal variation was much bigger (>1000 DNs in 10 min). Smigaj et al. [50] witnessed a spike (approximately 1.5 °C) within the first 15 min when imaging a blackbody at 25 °C with Optris PI-450. Berni et al. [9] also noticed the convergence in measurements to the blackbody temperature (40 °C) using FLIR Thermovision A40M. The observations were initially overestimated by nearly 2 °C and gradually approached the blackbody temperature with a decreasing rate of change. It is, therefore, noteworthy that the pattern and extent of changes will depend on the choice of a camera. 

The issue of automatic NUCs has also been taken into consideration. Previous research dealt with the calibration of miniaturized TIR camera models by setting the NUC execution to manual mode [39,47,51]. The influence of automated NUCs was rarely mentioned. When using any miniaturized TIR camera model, it is advisable to examine to what extent the actual effect matches the claimed effect by the manufacturer. For many models, NUCs are automatically executed at a specified time interval or after capturing a certain degree of temperature change in the field of view (FOV). The effectiveness of NUC execution is only based on the temperature of the shutter. In an ideal situation, the assumption is true that the shutter temperature is the same as the rest of the camera [30]. However, such assumptions can no longer be valid in real-world tests. Even though NUCs can be optimized in a controlled environment, the temperature of the camera’s shutter, interior components, and lens cannot always be kept consistent. During flight campaigns, it is likely that the camera experiences dynamic changes in environmental variables such as ambient temperature, wind, or solar radiation. These changes can bring uncertainty to the temperature difference between camera parts, and the speed of the change may be faster than the execution frequency of NUCs. Accordingly, the effectiveness of NUCs would be significantly affected outdoors. In the tests of WIRIS, it was found that the automated NUCs introduced high uncertainty to the measured temperature. When the NUC time interval was long (30 min), the difference in signal response between FPA units showed a continuously increasing trend in the STD; after shortening the time interval to 2 min, the signal response consistency between FPA units was high and remained stable during the 2-h test. Considering both the manufacturer’s recommendation and the results of this experiment, the higher frequency NUC is recommended. The problem to face in this choice is the frequent and large variations of the measurement signal. Ideally, the NUC keeps the response signals of each FPA unit consistent [28]. In this process, the gain and offset for all microbolometers on the FPA are adjusted to ensure that [52]. Specifically, after each NUC update, temperature drifts cause the output signal deviation of different FPA units to vary in size and direction over time, but the output signal’s change pattern of each FPA unit should be fixed. In this way, the changing pattern of the overall average output should still be similar. However, we still suggest conducting quantitative research on the influence patterns of the adopted TIR camera’s NUC on measurement results in different environments. 

The non-uniformity problem affects the performance of TIR imaging systems and their application in many domains. The frequently used strategies to solve this problem are as follows: one is to develop new materials for camera components to reduce the non-uniformity of the device [53]; the other is to use signal processing to correct the problem [8]. Signal processing can help extract noise-free signals from long-sequence TIR images. In the stabilization test (Section 3.1), the time-domain signal (such as the relationship between the measured temperature and time) still contains significant noise. After using the first-order difference to analyze the time-domain signal, the changing curve of the temperature measurements is presented with smoothing applied. It is easier to judge the overall trend of the temperature development (Figure 3). However, due to different sources of noise in the TIR sensor circuit, the measurement results usually contain these random signals from external interference and random factors such as thermal effects. As shown in the research of Kelly et al. [37] and Aubrecht et al. [8], frequency-domain analysis using fast Fourier transform (FFT) can help classify the noise generated by different frequencies and filter out the noise in a specific frequency spectrum. Therefore, the signal-to-noise ratio in the frequency-domain signal is small, and it is easier to extract the real signal with less distortion.

The influence of the sensor temperature on the measurement cannot be underestimated. In this study, since the FPA temperature cannot be obtained and manually adjusted, its effects were tested indirectly. When the ambient temperature changes, the FPA temperature should change in the same direction, but the magnitude of the change differs. Therefore, the influence of the sensor temperature on the measurement can be indirectly characterized by the correlation between the ambient temperature and the measurement. In addition, different degrees of influence can be seen from the experimental results of different temperature blackbodies. In the research of Lin et al. [39], the relation between the FPA temperature, housing temperature, and the measured temperature is depicted, before and after applying its non-uniformity correction methods. As the housing temperature highly correlates with the ambient temperature, the similarity between the change of FPA temperature and housing temperature suggests the high correlation between the sensor temperature and ambient temperature. In addition, another practical difference exists in generating the blackbody curves. Instead of depicting the relationship between the measured signal and blackbody temperature for different blackbody temperatures, we presented the correlation between the ambient temperature and measured temperature for the available blackbody temperatures. This change gives the opportunity to still quantify the ambient temperature (led to the difference in camera’s sensor temperature)’s impact on outputs when having no blackbodies with multiple temperatures. 

The measuring distance’s influence can be translated into the atmosphere’s interference. According to Ribeiro-Gomes et al. [42], the influence of distance on measurements should not be ignored. As this study indicated, the measured temperature was negatively correlated with the measuring distance. As can be seen from Figure 9 and Figure 10, the radiometric measurements suggested that the blackbodies appeared several Celsius degrees colder than the actual target temperature when those were 4 m away from the sensors. However, other factors could also have contributed to the uncertainty. The varying measuring distance causes the change of the atmospheric interference between the sensor and the target object. As a result, the signal proportion of the measured object differs in the acquired imagery. In earlier practices, Chrzanowski [54] investigated the influence of the difference between the sensor-target distances under laboratory calibration and real-world applications on the accuracy of the temperature measurements with IR imaging systems. The results have shown that the inaccuracy expanded significantly due to this source. Zhang et al. [55] measured different temperatures of blackbodies (50 °C, 150 °C, 250 °C, and 500 °C) at varying distances (0–15 m) in the controlled laboratory environment with a TIR sensor. The measured error was less than 0.7 °C for all sessions, which was much smaller than the results in this study. The author also successfully applied a correction model to eliminate the measuring distance’s influence to less than 0.1 °C. The model considers major aspects of influence: the angular field of the TIR imager, the difference between the target object’s temperature and ambient temperature, and the atmospheric transmittances caused by measuring distance. It is uncertain whether this modeling practice can be extrapolated to field tests, as the real-world situation brings more disturbances to the measuring process [56]. Generally, the temperature retrieval process must include an atmospheric correction in satellite remote sensing, while insufficient attention has been paid to this in the UAV-based research [57]. Applying atmospheric correction knowledge can help eliminate the ambient environmental condition’s influence on UAV-based temperature measurements at varying flight heights. 

In previous practices, the fixed pattern noise of the TIR cameras and the change of vignetting effects over time was monitored by completely covering the FOV of the cameras with a blackbody. The temperature differences between pixels in the center and at the edge of the sensor can reach up to more than 2 °C [37]. This problem arises in the ortho-mosaic creation based on collected UAV images [58]. There are a variety of methods used for correcting vignetting effects [59,60,61]. Instead of correction solutions, it is also recommended to only trust a certain portion of the central area in the frame during the data collection and analysis procedures. 

Based on the experimental results, the following attention points should be considered before actual field practices of miniaturized TIR cameras:

1. A larger extent of temperature shifts has been witnessed directly after activation. We suggest allowing a longer time for UAV-mounted cameras to stabilize after activation, preferably at least one hour. For handheld devices, a stabilization period of 30–40 min is enough. Other studies suggest 30–60 min to stabilize whether or not the same abrupt changes have been observed in the beginning [22,34,43]. There is a trade-off between the duration of the stabilization period and the length of the UAV flight.
2. For cameras of which the automatic corrections are executed periodically, there is a trade-off between ensuring the data consistency and diminishing temperature drifts in flight campaigns. If the former is chosen, then longer-period NUCs will make a larger sequence of collected imagery comparable to each other upon using drift correction methods. Otherwise, image mosaic will be a major problem with the choice of frequent NUCs. For UAV-based cameras, we are inclined to still suggest high-frequency NUCs if the acquired TIR imagery is applied to quantitative applications. It is best to perform NUCs with the smallest feasible time interval one can choose from the camera settings. For handheld cameras, it would be better to always capture images shortly after manual or automated NUCs to avoid drifts.
3. In the laboratory experiment, following factors contributed to the accuracy changes in distance tests: (a) noise as a result of radiation from other objects in the room; (b) water vapor absorption (this study had very high humidity settings); (c) size of the blackbody. For all miniaturized TIR cameras, the temperature measurements are underestimated to a larger extent as the measuring distance increases. Tests on different flight heights before actual flight campaigns can provide insight into the influence of atmospheric interference in the fields. Based on the test results, the researcher can prepare formal experiments with the corresponding influence degree in mind. Afterwards, suitable atmospheric correction models could be used to effectively reduce the deviation of observation values caused by atmospheric interference as an option.
4. The measured temperature is highly correlated with the sensor temperature’s variations. Thus, real-time observations of the sensor temperature (or FPA temperature) are preferred in flight campaigns if applicable for specific camera models (e.g., FLIR Tau 2). This can contribute to the calibration of measured temperature in the post-processing procedure.
5. Large fluctuations in camera signal have been found during the treatments of testing wind and heating on camera performance. Cameras can be shaded during flights to diminish this disturbance.
6. Previous studies concluded that it is not feasible to directly translate the laboratory calibration methods into field tests as the uncertainty expands to a much larger extent. However, as this study has demonstrated, a laboratory-based simulation approach quantifies the inaccuracy in measured temperature brought by varying influencing factors, which can guide field experimental set-ups. It still needs to be explored how the problems found in temperature measurements can be avoided in an operational context where all influencing factors add up by continuing follow-up field tests.

## 5. Conclusions

This research assessed the effects of multiple sources of influencing factors on the performance of two radiometric miniaturized TIR camera models. Concerning the influence of the cameras’ intrinsic characteristics, the laboratory experiments in a climate room suggest that the duration of the warm-up period may vary among different models. However, 30 min for handheld cameras and 60 min for UAV-mounted cameras can guarantee acceptable measurement accuracy. During measurements, the seemingly random changes that automatic NUCs may bring to the temperature observations should not be neglected. A suitable time-interval setting of NUCs, together with drift correction methods can compensate for the data consistency problem in image mosaics. In addition, it is recommended to contact the manufacturers to better understand the NUC’s effects based on the differences between the factory calibration and user tests (without a complete series of precise instruments for calibrating). To eliminate the effect of noises, it is recommended to transform measurements into the frequency-domain signal for diminishing the signal-to-noise ratio. The variation in sensor temperature also has a negative influence on measurement accuracy. Lastly, ambient environmental conditions should be taken into account in the experimental design of field tests. The results clearly show the non-negligible influence of wind, radiation, and atmospheric interference in distance tests. In the fields, the measurement uncertainty may expand to several degrees if these factors are not properly considered.

## Figures and Tables

**Table 1:** The technical specification of used miniaturized thermal infrared (TIR) cameras.

Camera	Workswell WIRIS 2nd GEN 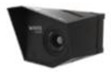	FLIR E8-XT 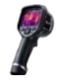
TIR Resolution	640 × 512 pixels	320 × 240 pixels
Field of View (FOV)	69° × 56° (focal length = 9 mm)	45° × 34°
Active sensor size of focal plane array (FPA)	1.088 × 0.8705 cm	-
Temperature ranges	−25 °C to +150 °C, −40 °C to +550 °C	−20 °C to +550 °C
Temperature sensitivity	0.05 °C (50 mK)	< 0.05 °C (50 mK)
Accuracy	±2 °C or ±2% of reading	±2 °C or ±2% of reading, for ambient temperature 10 °C to 35 °C
Spectral Range	7.5–13.5 μm	7.5–13.0 μm
Calibration	Yes	
Detector type	FPA, uncooled vanadium oxide (VO_x_) microbolometer	

**Table 2:** Overview of environmental parameter settings related to sets of experiments.

Experiment	TIR Camera	Measuring Distance	Capture Time Interval /Total Time	Time Interval of Non-Uniformity Corrections (NUCs)	Atmospheric Temperature (T_a_)	Relative Humidity (RH)
a. Stabilization time	WIRIS	0.5 m	5 s/2–3 h	2 and 30 min	15 °C	70%
	FLIR E8-XT		5 s/1 h	turned off		
b. Ambient temperature’s influence	WIRIS	0.2 m	30 s/ 6–19.5 h	30 min	Setting points: 5 °C, 10 °C, 14 °C, 18 °C, 20 °C, 22 °C, 25 °C; the actual temperature change is continuous, approximately from 5 °C to 25 °C	
	FLIR E8-XT		30 s/5.5 h	turned off		
c. Distance’s influence	WIRIS	0.5 m, 1.0 m, 2.0 m, 4.0 m	5 s/80 min	30 min	15 °C	
	FLIR E8-XT			turned off		
d. Wind and heating-up effects	WIRIS	0.5 m	5–10 s /2–3 h	30 min		

## Data Availability

The datasets used in this study are available upon request.

## Supplementary Materials

The following are available online at https://www.mdpi.com/article/10.3390/s21248466/s1. Figure S1: The time series curves of WIRIS camera readings, housing (surface) temperature measurements from one resistance temperature detector (RTD) data logger, ambient temperature measurements provided by the laboratory. The camera readings have been recorded against a blackbody calibrator at 35 °C, over 2 h after activation. The development of the measured temperature measurements’ standard deviation (STD) across pixels is also presented. Figure S2: The measured average temperature of the blackbody calibrator (35 °C) area, as recorded by FLIR E8-XT over 1 h after switching on the camera. The housing temperature’s development of the camera is also depicted. Figure S3: Simulation of the effect of wind on WIRIS while imaging a blackbody at 35 °C with two levels of wind speeds (lower—2.7–3.1 m/s, higher—2.9–3.3 m/s). A one-hour control period was set between wind tests. The housing temperature’s development (of the camera) and the STD of the measured temperature are also included. From approximately 48 min to the end of the session, there was a malfunction of NUC.
