# Supplementary material for: Factors Influencing Temperature Measurements from Miniaturized Thermal Infrared (TIR) Cameras: A Laboratory-Based Approach
Source: Sensors (Basel). 2021 Dec 18;21(24):8466. doi: 10.3390/s21248466 (PMC8706234; doi:10.3390/s21248466)
Supplement: Supplementary file 1 [file sensors-21-08466-s001.zip › sensors-1497384-supplementary.pdf]

Supplementary Materials

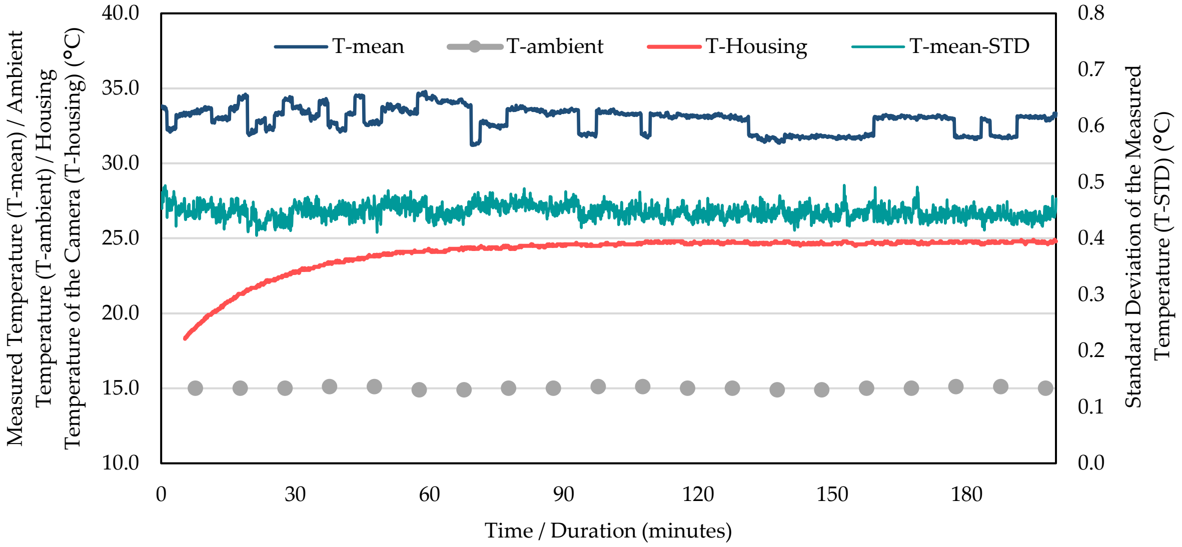

Figure S1. The time series curves of WIRIS camera readings, housing (surface) temperature measurements from one resistance temperature detector (RTD) data logger, ambient temperature measurements provided by the laboratory. The camera readings have been recorded against a blackbody calibrator at 35 °C, over 2 hours after activation. The development of the measured temperature measurements' standard deviation (STD) across pixels is also presented.

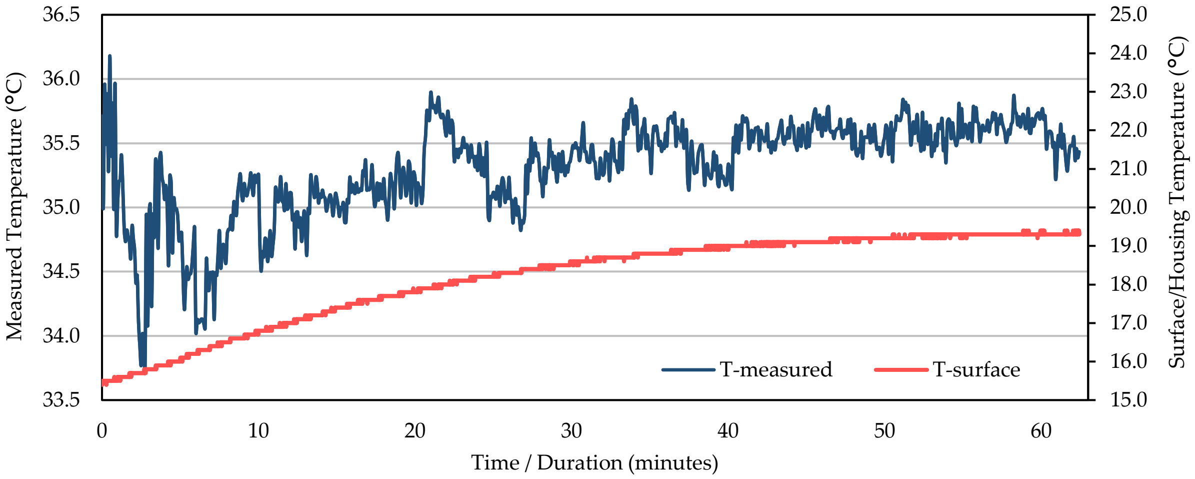

Figure S2. The measured average temperature of the blackbody calibrator (35 °C) area, as recorded by FLIR E8-XT over 1 hour after switching on the camera. The housing temperature's development of the camera is also depicted.

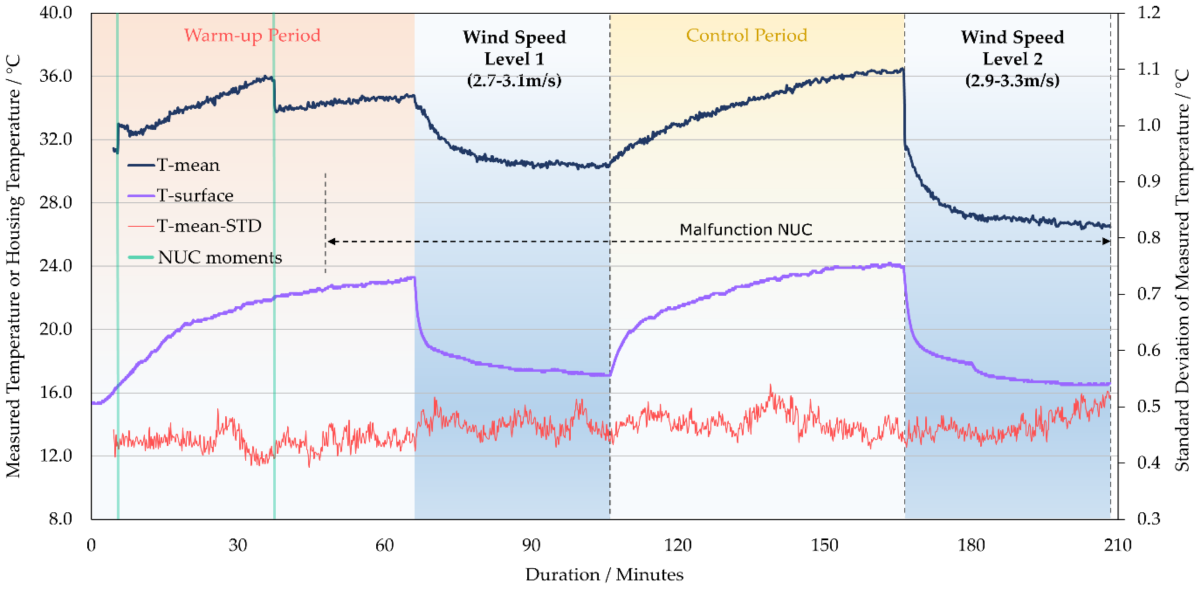

Figure S3. Simulation of the effect of wind on WIRIS while imaging a blackbody at 35 °C with two levels of wind speeds (lower - 2.7-3.1 m/s, higher - 2.9-3.3 m/s). A one-hour control period was set between wind tests. The housing temperature's development (of the camera) and the STD of the measured temperature are also included. From approximately 48 minutes to the end of the session, there was a malfunction of NUC.
